# A review of Suicide Behavior Among Arab Adolescents

**DOI:** 10.1100/tsw.2005.84

**Published:** 2005-08-26

**Authors:** Mohammed Morad, Efrat Merrick, Amir Schwarz, Joav Merrick

**Affiliations:** ^1^ Clalit Health Services, Faculty of Health Sciences, Ben Gurion University of the Negev, Beer-Sheva, Israel; ^2^ Division for Community Health, Faculty of Health Sciences, Ben Gurion University of the Negev, Beer-Sheva, Israel; ^3^ Center for Multidisciplinary Research in Aging, Faculty of Health Sciences, Ben Gurion University of the Negev, Beer-Sheva, Israel; ^4^ National Institute of Child Health and Human Development, Faculty of Health Sciences, Ben Gurion University of the Negev, Beer-Sheva, Israel; ^5^ Division for Mental Retardation, Ministry of Social Affairs, Jerusalem, Israel; ^6^ Office of the Medical Director, Ministry of Social Affairs, Jerusalem, Israel

**Keywords:** Adolescence, suicide, Islam, human development, holistic health, public health, Israel

## Abstract

Islam prohibits the taking of one's life, because this way you will interfere with the work of G-d (Allah), which is clear from several places in the Quran. Concerning individual suicide or suicide attempts in various Arab countries the literature is sparse and the incidence low. In this paper we present a review of research from Israel showing that suicide epidemiology among the Arab population of children and adolescents display a low incidence, but an increase has been observed over the past decade, but still much lower than the Jewish population. We believe that there is a need for the development of prevention and intervention strategies in order to keep this incidence low.

## INTRODUCTION

If the body is destroyed, instead of being embalmed according to the Egyptian tradition, the soul will loose the house that it is supposed to return to every night in order to be renewed and reborn every morning upon sunrise, thereby living eternally[1]. Islam is guided by the verses: “Nor take life—which G-d has made sacred—except for just cause”.(Al-Israa' 17:33), and “If anyone killed a person not in retaliation for murder (without just cause), or to spread mischief in the land, it would be as if he killed all mankind, and if anyone saved a life, it would be as if he saved all mankind.”(Al-Ma'idah 5:32) So, if someone was to prevent a person from committing suicide it is as if he has saved the life of the whole of humanity, which interestingly is a philosophy close to that of Judaism.

God tells us that he gives life and takes life as written in the Quran: “Have you not turned your gaze to one who disputed with Abraham about his Lord because G-d granted him power?” Abraham said, "My Lord is He who gives life and death." (Al-Baqarah 2:258) Life is given by G-d to each person for a purpose and we are here on earth to fulfill that purpose. We are therefore not free to decide when our purpose is finished and when we should die. If we take or attempt to take our own life, we are trying to perform a function that belongs to G-d. The Quran states: “Nor kill nor destroy yourselves, for surely God has been to you Most Merciful.”(An-Nisaa' 4:29) In

Islam it is believed that a person who takes his own life will be tormented as the Prophet said: "He who commits suicide by throttling shall keep on throttling himself in the Hellfire (forever), and he who commits suicide by stabbing himself shall keep on stabbing himself in the Hellfire." (Al-Bukhari Volume 2, Book 23, Number 446) Thabit bin Ad-Dahhak narrated: The Prophet said, "… … whoever commits suicide with piece of a iron will be punished with the same piece of iron in the Hellfire.", and "A man was inflicted with wounds and he committed suicide, and so Allah said: My slave has caused death on himself hurriedly, so I forbid Paradise for him." (Al-Bukhari Volume 2, Book 23, Number 445).

Going into war knowing with certainty that one will die is not suicide (intihar), but martyrdom (istishhad) and therefore a form of self-sacrifice in the path of G-d, or a way to win the eternal affection in order to enter paradise, which has been discussed presently in reference to suicide bombings. This is not the issue for this chapter, but rather individual suicide.

Religion is a very important component in the Arab culture, but there are other multiple social, spiritual, physical and financial tensions that affect suicidal behavior of adolescents, which may be the reason for the recent change in trends of adolescent suicide seen in the Arab population in Israel.

## RESEARCH ON SUICIDE IN ARAB COUNTRIES

A search in Medline/Pubmed (http://www.pubmedcentral.nih.gov/) on “suicide and Arabs”, or “suicide and Islam” revealed very few entries.

One study from Kuwait during 1984-1985[2], with a consecutive sample of 92 parasuicides aged 14-44 years, showed that self-poisoning accounted for 85.9%. The majority (58.3%) were expatriates who were mostly males, while the minority (41.7%) were Kuwaiti nationals. Family discord was the dominant antecedent life event in Kuwaitis, while non-Kuwaiti Arabs and non-Arab expatriates reported an excess of work-related problems as precipitating factors. Non-Arab females resorted to more violent methods than other females. Acute situational distress was the most common diagnosis, while depression was present in about 25% of the total sample.

A study from Jordan[3] investigated the impact of national and religious events on the rate of parasuicide and compared the number of reported parasuicides during the month of Ramadan with the month before and after Ramadan in Jordan for the years from 1986 to 1991. Significantly fewer parasuicides were reported during Ramadan than in either the month preceding,, or the month following Ramadan.

Turkey, an Islamic country, reviewed the medical records of 185 cases of suicide attempts referred to a psychiatric department[4]. There was a predominance of single females (students, housewives, and employees) with drug overdose as the most common method of suicide attempt and depression as the most common diagnosis. The repetition rate of suicide attempt was 43.3%.

Risk factors of overdose among adolescents 15-24 years of age was studied at the psychiatric hospital in Bahrain[5]. All those who attempted suicide in the country during an 18-month period were identified and a hospital-matched control was selected for each case. There were106 cases, but six females refused an interview. The overdose attempter was more likely than the control to be unemployed, a member of a non-intact family, having a mother whose education was high school or above, not having a friend, involved in a boy/girlfriend relationship and a cigarette smoker. More students among attempters had failed an examination in the past year than matched controls. Stress generated from living in a non-intact family, interpersonal relationships mainly with the opposite sex, unemployment and school performance came out as the main risk factors.

The Department of Forensic Medicine in Dubai, United Arab Emirates found an annual rate of suicide in Dubai for the period 1992-2000 at 6.2 per 100,000[6]. There was a non-significant peak incidence in November, while July recorded the lowest incidence, and Fridays showed the lowest incidence with no significant difference between Muslims and non-Muslims. Victims were predominantly male expatriates with no significant difference between Muslims and non-Muslims. The majority of victims were of the age group 21-40 years of age with a significant increase in teenage females. Hanging was the commonest method for committing suicide, while females and non-Indian expatriates resorted significantly to jumping and self-poisoning. The age of the victim had no effect on the choice of the method used to commit suicide. The majority of incidents took place in the victim's own house. History of psychological illness or trauma was available in 9.7% of cases with depressive illness and recent unemployment as the major triggers for suicidal impulse.

## TRENDS IN SUICIDE IN ISRAEL 1975-1989

Data from the Central Bureau of Statistics (CBS) in Israel over a 15 years period (1975-1989) was used to examine sociodemographic correlates and trends in suicide among young Jews, Moslem Arabs, Druzes, and Christian Arabs in Israel[7]. Among Jews, gender differences were found in the army-age group where males committed suicide more frequently than females (OR = 3.2; 95% CI 2.2, 4.6). A similar result was obtained for rates of suicide combined with undetermined external causes (UEC) of death for both the young adolescents (OR = 2.0; 95% CI 1.3, 3.2) and the army age groups (OR = 4.9; 95% CI 3.7, 6.5), while among the young Moslem Arabs and Christian Arabs, gender differences for both types of rates were not found. In the Druze, all deaths in the army age group recorded as suicide, or UEC of death, were among males[7].

Among children, rates of suicide combined with UEC causes of death were statistically higher among non-Jews than Jews. No statistical differences were found between the various national/religious groups for suicides only. Among the young adolescents, the rates were also significantly higher in the Moslem Arabs and Druze than in Jews for suicide combined UEC of death. Male Druzes had higher rates than both Jewish and Moslem Arab males (OR = 2.8; 95% CI 1.0, 7.6), and when suicides alone were examined, Druzes were the only group to have higher rates than Jews (OR = 2.8; 95% CI 1.0, 7.7), a result of an increased risk among male Druzes (OR = 3.4; 95% CI 1.0, 11.1)[7].

In the army age group, the differential pattern among the national/religious groups began to reverse toward that found among the adult population in which rates were higher for the Jews. For both sexes combined, statistical differences in this age group were found only for the comparison between Jews and Moslem Arabs for suicide combined UEC of death. Jewish males had elevated rates of both suicide combined UEC of death and suicide (OR = 2.6; 95% CI 1.4, 5.0) compared with Moslem Arab males. In contrast, among females, Moslem Arabs had higher rates of suicide combined with UEC of death. Army age male Druzes had higher rates of suicide combined with UEC of deaths than Moslem Arab males (OR = 3.5; 95% CI 1.5, 7.9) and higher rates of suicide than Moslem Arab males (OR = 5.2; 95% CI 1.9, 14.3) and Christian Arab males (OR = 8.2; 95% CI 1.0, 66.6)[7].

It therefore seems that service in the army has an effect among male Druze and Jews. The Jewish males in the army age group had a marked increase in their risk of suicide combined with UEC of death (OR = 5.1; 95% CI 3.8, 6.8) and suicide (OR = 3.8; 95% CI 2.6,5.5) compared with the young adolescent group. The army age group was also at a higher risk for suicide combined with UEC of death compared with individuals who had just completed army service, aged 21-29 years (OR = 1.6; 95% CI 1.4, 1.8) and at equal risk for suicide (OR = 1.0). The same, but not statistically significant, was found for the male Druzes; compared with young adolescents there was an OR = 1.8, and contrasted to those who just completed army service there was an OR = 1.5 for suicide combined with UEC. As for suicide, army age male Druzes had an OR = 2.2 compared with young adolescents and an OR = 1.3 in contrast to 21-29 year olds[7].

The investigation of trends for the rates of suicide combined with UEC of death, 1975-1989, was explored among males and females both separately and jointly, but the analysis was limited to Jews and Moslem Arabs due to the small number of suicides among the other groups. Among children, the risk for death by suicide combined with UEC of death appeared to be increasing for Jews (beta = .08, SE = .04, $p < .04$) and Moslem Arabs (beta = .12, SE = .04, $p <.003$). For Jews, this increased risk was primarily due to the females (beta = .29, SE = .10, $p < .03$). For Moslem Arabs, both males (beta = .09, SE = .05, $p < .05$) and females (beta = .17, SE = .07, $p < .02$) had rising rates of suicide combined with undetermined causes of death. However, when suicide rates were examined excluding UEC of death, the rates fell significantly among Jewish children (beta = -.14, SE = .07, $p < .05$) over the 15-year period, in particular among Jewish males (beta = -.22, SE = .10, $p < .02$). In the young adolescent group, only Jews showed a temporal change in the rates of suicide combined with UEC of death (beta = .05, SE = .03, $p <.04$). This increase was in male deaths (beta = .07, SE = .03, $p < .04$) and it resulted in an overall rising rate for the group. A similar trend was seen for suicide alone (beta = .07, SE = .03, $p < .03$) and for male Jews (beta = .11, SE = .04, $p < .02$). The army age group among the Jews, however, had decreasing rates of suicide combined with UEC of death (beta = -.07, SE = .013, $p < .0001$) accounted for by the pattern found among the males (beta = -.08, SE = .01, $p < .0001$). When only suicide was examined for army age Jews, no overall change in the rates over the 15 years studied was observed. Among Moslem Arabs, no overall change was seen in suicide combined with UEC of death for the 18-20 year olds. There were, however, gender differences where Moslem Arab males had an increase in rates with time (beta = .18, SE = .07, $p < .005$), while females showed a decrease (beta = -.18, SE = .05, $p < .0006$). Female Moslems also showed a decrease in suicide when examined without undetermined causes of death (beta = -.27, SE = .08, $p < .002$)[7].

From this study[7], it could be concluded that suicide among children seemed to have increased over the 1975-1989 period, especially among Moslem Arabs, while for young adolescents the increase was noted in the Jews. For the army age Jewish group, the combined rates clearly showed a decline, an effect resulting more from the declining rates of the UEC of death than from the suicide rates. The combined rates for Moslem Arabs in the 18-20 year old group remained rather stable, but with gender differences. The rates of suicide among the 15-19 year old Israeli Jews (5.3 per 100,000 males, 1.9 per 100,000 females) and Moslem Arabs (2.7 per 100,000 males, 2.8 per 100,000 females) were among the lowest in the world, but when UEC of death is taken into account, then the Israeli rates begin to approach those of other nations (Jews: 10.7 per 100,000 males, 2.9 per 100,000 females; Moslem Arabs: 5.5 per 100,000 males, 6.5 per 100,000 females)[7].

## TRENDS IN SUICIDE IN ISRAEL 1984-1994

Another study looked at the trends in suicide incidence rates in Israel from 1984-1994[8] in order to identify sub-populations at high risk for suicide and to identify suicide methods associated with increased risk using the computerized data files of the Israeli Central Bureau of Statistics, which includes cause of death and sociodemographic variables.

Suicide rates were higher for Jews than for Arabs and higher for men than for women for the whole population. The rates among both population and sex groups increased directly with age. A significant increase over the years studied was found for Jewish men, particularly in the 18-21 year old age group, with a marked increase in the use of firearms. The average suicide rate for 1984-1994 was 8.7 per 100,000 (increased from 7.1 in 1984-85 to 9.3 in 1992-94) and when UEC (undetermined external causes) of death were included, the average increased to 12.6 (9.8 in 1984-85 and 12.5 in 1992-94)[8]. Israeli Jews were more than three times at risk for suicide as compared to Arabs (9.8 versus 2.9) and this difference was still evident, but smaller, when UEC were included (13.7 versus 7.2). The suicide rate for Jews versus Arabs was 3.1 for males and 4.4 for females. When looking at the trends over time, there was an increase for Jewish males from

11.0 in 1984-1985 to 15.9 in 1992-1994 (Arab males 2.6 to 3.9), an increase for Jewish females from 5.1 to 5.6 and a decrease from 1.6 to 1.5 for Arab females[8].

In the 10-14 year age group, the average suicide rate was 0.5 per 100,000 (1.0 including UEC), 2.8 for the 15-17 year olds (4.6 with UEC) and 6.7 for the 18-21 year olds (10.8 with UEC). For this last age group the increase over time was marked with 3.9 for 1984-1985 to 18.2 in 1992-1994, (from 10.5 to 21.8 if including UEC).

## INTENTIONAL INJURIES AMONG CHILDREN AND YOUTH

In order to examine the incidence and outcome of intentional injuries requiring emergency room care among children and adolescents, a study was performed in 1994 with the population of 0-17 year olds who presented to 23 out of a possible 28 emergency rooms all over the country during that one year period[9]. A 6% to 9% random sample of days was selected at each hospital, and for each selected day the relevant records were reviewed for cause, nature, and outcome of injuries and sociodemographic information.

The annual incidence for intentional injuries resulting in emergency room visits was 19.6 in 10,000 children and adolescents aged 0-17 years of age (95% confidence interval (CI) 17.4-21.8 in 10,000). Fights/assaults constituted 54.1% of the presentations, abuse and rape 10.3% and self-inflicted injuries 10.8%. The rates were higher among boys than girls for fights/assaults and abuse, whereas attempted suicide and rape were three times higher among girls than boys. Nearly twice as many Jewish children and adolescents presented to the emergency room for intentional injuries than Arab children and adolescents, with the ratio becoming even greater for attempted suicide. Of all the intentionally injured, 21.7% were hospitalized. The mortality rate was 1.1 in 100,000 (95% CI =.7-1.7/100,00) with no significant gender difference observed. No cases of suicide were reported for the Arab population, but there were 30 suicide attempts (rate of 0.6 per 10,000)[9].

## CONCLUSIONS

Suicide and suicide attempts occur in the Arab Israeli population of children and adoloscents, but at much lower rates than the Jewish population, which is again lower than in the Western population. According to both Judaism and Islam, suicide is condemned and not an accepted behavior, but in recent years an increase has been observed and especially in the 18-21 year age group with an increase in firearm related suicide. There is a need for professionals working with the Arab adolescent population to be more aware of this problem and to build strategies for prevention and intervention.

## References

1. Okasha, A. Mental health in the Middle East: *An Egyptian perspective*. (1999) *Clin. Psychol. Rev.***19**(8), 917-933.
2. Suleiman, M.A., Nashef, A.A., Moussa, M.A., and el-Islam, M.F. (1986) Psychosocial profile of the parasuicidal patient in Kuwait. *Int J Soc Psychiatry*. **32**(3), 16-22.
3. Daradkeh, T.K. Parasuicide during Ramadan in Jordan. (1992) *Acta Psychiatr Scand*. **86**(3), 253-254.
4. Cosar, B., Kocal, N., Arikan, Z., and Isik, E. (1997) Suicide attempts among Turkish psychiatric patients. *Can J Psychiatry*. **42**(10), 1072-1075.
5. Al Ansari, A.M., Hamadeh, R.R., Matar, A.M., Marhoon, H., Buzaboon, B.Y., and Raees, A.G. (2001) Risk factors associated with overdose among Bahraini youth. *Suicide Life Threat. Behav*. **31**(2), 197-206.
6. Koronfel, A.A. (2002) Suicide in Dubai, United Arab Emirates. *J Clin Forensic Med*. **9**(1), 5-11.
7. Kohn, R., Levav, I., Chang, B., Halperin, B., and Zadka, P. (1997) Epidemiology of youth suicide in Israel. *J Am. Acad. Child Adolesc. Psychiatry*. **36**(11), 1537-1542.
8. Lubin, G., Glasser, S., Boyko, V., and Barell, V. (2001) Epidemiology of suicide in Israel: A nationwide population study. *Soc. Psychiatry Psychiatr. Epidemiol*. **36**, 123-127.

9. Gofin, R., Avitzour, M., Haklai, Z., and Jellin, N. (2000) Intentional injuries among the young: Presentation to emergency rooms, hospitalization and death in Israel. *J. Adolesc. Health*. **27**, 434-442.

**Handling Editor:**
Hatim A. Omar, Associate Editor for *Child Health and Human Development* --- a domain of *TheScientificWorldJOURNAL.*

## BIOSKETCHES

**Mohammed Morad,** MD, is specialist in family medicine, a lecturer in family medicine affiliated with the National Institute of Child Health and Human Development, Division of Community Health and Center for Multidisciplinary Research in Aging, Faculty of Health Sciences, Ben Gurion University of the Negev and the medical director of a large area clinic in the city of Beer-Sheva operated by the Clalit Health Services. Publications on Bedouin health, health aspects, spiritual health and aging in persons with intellectual disability, and a presenter on topics like health policy and services for the disadvantaged at national and international conferences. E-mail: morad62@013.net.il

**Efrat Merrick,** is a medical student at the Sackler School of Medicine, Tel Aviv University and a research assistant at the National Institute of Child Health and Human Development, Faculty of Health Sciences, Ben Gurion University of the Negev, Beer-Sheva, Israel. E-mail: efratmerrick@gmail.com

**Amir Schwarz, MSW, PhD is** the chief social worker, Division for Mental Retardation, Ministry of Social Affairs, Jerusalem, Israel. E-mail: siim@zahav.net.il

**Joav Merrick, MD, DMSc,** is professor of child health and human development affiliated with the Center for Multidisciplinary Research in Aging, Zusman Child Development Center, Division of Pediatrics and Community Health at the Ben Gurion University, Beer-Sheva, Israel, the medical director of the Division for Mental Retardation, Ministry of Social Affairs, Jerusalem, the founder and director of the National Institute of Child Health and Human Development. Numerous publications in the field of child health and human development, rehabilitation, intellectual disability, disability, health, welfare, abuse, advocacy, quality of life and prevention. Received the Peter Sabroe Child Award for outstanding work on behalf of Danish Children in 1985 and the International LEGO-Prize (“The Children’s Nobel Prize”) for an extraordinary contribution towards improvement in child welfare and well-being in 1987. E-Mail: jmerrick@internet-zahav.net. Website: www.nichd-israel.com
